# Genomic Aberrations of Antisense Gene Transcripts in Head and Neck Cancer

**DOI:** 10.3390/cells15010009

**Published:** 2025-12-19

**Authors:** Jishi Ye, Stacy Magdalene Abbang, Yuen-Keng Ng, Vivian Wai Yan Lui

**Affiliations:** 1 Department of Pain Management, Renmin Hospital of Wuhan University, Wuhan 430060, China; 2 Biochemistry and Cancer Biology Program, Department of Biochemistry and Molecular Biology, Medical College of Georgia, Augusta University, Augusta, GA 30912, USA; 3 Department of Medicine, Medical College of Georgia, Augusta University, Augusta, GA 30912, USA; yng@augusta.edu; 4 Georgia Cancer Center, Augusta University, Augusta, GA 30912, USA; 5 Department of Biochemistry and Molecular Biology, Medical College of Georgia, Augusta University, Augusta, GA 30912, USA

**Keywords:** antisense genes, head and neck cancer (HNC), functional roles, biomarkers, drug sensitivity, signaling regulation

## Abstract

**Highlights:**

**What are the main findings?**

- Only 2.3% (52 total) of all human antisense genes have been examined in head and neck cancer (HNC) thus far.
- *HOXA10-AS*, *LEF1-AS1*, *MSC-AS1*, and *ZEB2-AS1* have clinical promise for future biomarker development.

**What are the implications of the main findings?**

- Robust cross-validations on antisense gene aberrations will promote biomarker development and implementation in the HNC field, as genetic biomarkers are lacking.
- Antisense gene aberrations and treatment responses are underexplored and warrant future studies.

**Abstract:**

Antisense genes (usually suffixed by -AS) represent a class of long non-coding RNAs (lncRNAs) transcribed from the opposite strand of annotated human genes or exon(s). A total of ~2236 human antisense genes exist in the human genome. Their genomic locations with respect to the corresponding sense genes, their dysregulated expression patterns in cancer specimens, and clinical associations with patient outcomes reveal their potential importance in clinical settings. As of today, there lacks a comprehensive review of HNC-associated antisense genes/transcripts to help move forward the antisense field for genetic biomarker development or future drug research. In total, 2.3% (52/2236 antisense genes) of all known human antisense genes have been investigated in head and neck cancer (HNC). Thus, we perform a comprehensive review of the genomic aberrations (mutations, copy number changes, RNA-expression dysregulation, and single nucleotide polymorphisms) associated with HNC patient prognosis, disease progression, cancer cell signaling, drug sensitivity, and radio-resistance. Four antisense genes, namely *HOXA10-AS*, *LEF1-AS1*, *MSC-AS1*, and *ZEB2-AS1*, have been clinically cross-validated and have consistently demonstrated to be associated with patient outcomes in multiple independent cohorts by different research teams, with clear evidence for the prioritization of clinical biomarker development in HNC. Single nucleotide polymorphisms (SNPs) of antisense genes with evidence for HNC risk or outcomes should be further validated in different ethnic groups, for potential global HNC applications.

## 1. Introduction

**Figure 1:**
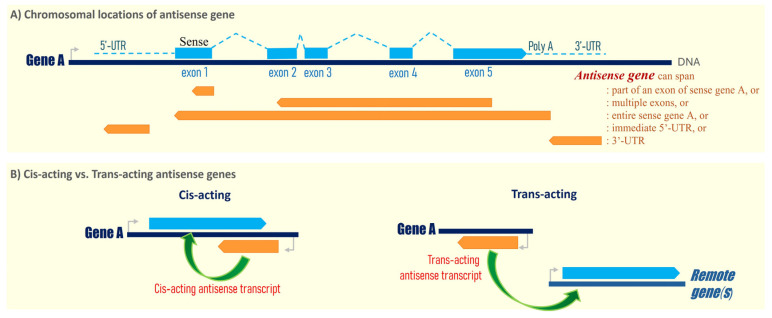
(**A**) Schematic showing the chromosomal location of a sense gene (gene A, for example) and the antisense gene found in human. Theoretically, an antisense gene runs in the opposite direction with sequence overlapping with either the exonic region(s) (e.g., overlapping with part of an exon, mostly exon 1; or overlapping with multiple exons), the 5′-untranslated region (5′-UTR) or 3′-untranslated region (3′-UTR) of its corresponding human gene. Sense gene transcript is shown with exons depicted as blue boxes, and introns in the blue dotted line. Orange arrows represent possible locations of the antisense gene (DNA). (**B**) Cis-acting vs. trans-acting antisense genes in gene regulation. A cis-acting antisense gene regulates the expression of its sense gene that overlaps or is immediately adjacent to the antisense gene, whereas a trans-acting antisense gene can regulate the expression of other gene(s) at different chromosomal location(s) in a remote manner. The antisense gene transcript is shown in orange arrow, while the sense gene transcript in light blue arrow.

Based on the most up-to-date HUGO Gene Nomenclature Committee gene list in 2025, there is a total of 2236 human antisense genes in the entire human genome (https://www.genenames.org/; accessed on 13 May 2025). All such antisense genes, standardized with the -AS, -AS1, or -AS2 suffixes (with a few exceptions), represent long non-coding RNA genes that are transcribed from the opposite strand of annotated human genes or exon(s). For example, the *CDKN2B-AS1* gene encodes a long non-coding RNA that is transcribed from the opposite (reversed) strand of the human *CDKN2B* gene, hereafter called the corresponding sense gene. Interestingly, among all the human antisense genes, some are found to span the entire opposite strand of the corresponding human gene, while some only encompass part of an exon or several exons, or encompass the immediate 5′- or the 3′-untranslated region (UTR) of a human gene. As the genomic locations of most antisense genes are universally situated in either the exons, 5′-, or 3′-UTRs of specific human genes (Figure 1A), antisense genes often distinguish themselves from another class of long non-coding RNA called LINCs (long intergenic non-coding RNAs), which are non-coding RNAs greater than 200 nucleotides long with no overlapping annotated coding genes.

As of today, it remains to be fully characterized if all 2236 human antisense genes are primarily involved in the regulation of their sense gene expression in cis. As defined, a cis-acting antisense gene regulates the expression of its sense gene that overlaps or is immediately adjacent to the antisense gene (Figure 1B). Studies have shown that some antisense genes can regulate exon-specific splicing, thus controlling the expression of various RNA isoforms, and can regulate the mRNA’s stability and location of the sense gene [1]. However, a trans-acting antisense gene can regulate the expression of other gene(s) at different chromosomal location(s) in a remote manner (Figure 1B). For example, some antisense genes can function as sponges for various cellular RNAs or microRNAs, thus regulating the expressions of multiple target genes simultaneously. Thus, both cis-acting and trans-acting antisense genes could potentially exhibit diverse cellular functions, and even global effects on cell growth, differentiation, and cell death, etc. The cancer field has begun to examine how human antisense genes contribute to human carcinogenesis, and whether they can serve as therapeutic targets for human cancers.

Head and neck cancer (HNC) is the sixth most common cancer in the world. The World Health Organization (WHO) projects > 1.08 million new cases per year by 2030 [2]. HNC encompasses cancers arising from the oral cavity, pharynx, and larynx. Etiologies include smoking, alcohol consumption, air pollution, and genetics, as well as infection with oncogenic viruses in specific parts of the pharynx, such as the Human Papillomavirus (HPV) for oropharyngeal cancer, and the Epstein–Barr virus (EBV) for nasopharyngeal cancer. Recent findings show that some human antisense genes are found to contribute to HNC tumorigenesis, cancer cell aggressiveness, and signaling regulation, as well as drug sensitivity. As of today, there lacks a comprehensive review of HNC-associated antisense genes/transcripts to help move forward the field for the development of genetic biomarkers or future drug research. Thus, we hereby summarize all recent clinical and biological findings on such 52 HNC-related antisense genes/transcripts, with the objective of identifying potential antisense genes as targets for future biomarker and drug development in HNC.

## 2. Only 2.3% of All Human Antisense Genes Explored in HNC

There is a rising interest in the study of human antisense genes in both normal and disease contexts. In the past decade, more than 2270 studies have been published on human antisense genes in human models and human tissues, as well as human cancers, based on the PubMed database. This has far exceeded the number of published studies (535) on “intergenic, non-exon-associated” long non-coding RNAs “LINCs”. Among all published human antisense gene studies, 61 studies on 52 antisense genes reported on HNC tumors or cell models (based on PubMed data captured on 13 May 2025). The 61 studies were identified using the keywords “antisense genes or transcripts” and “head and neck cancer” (including each HNC subtype each time, including oral, laryngeal, pharyngeal, salivary gland, oropharyngeal, or nasopharyngeal cancer) using the PubMed literature search engine on https://pubmed.ncbi.nlm.nih.gov/. All extracted information was cross-checked by two or all three authors of this review.

From a total of 2236 human antisense genes annotated by HuGO (listed in Supplementary Table S1), only approximately 2.3% of them have been studied in HNC (52/2236 genes). In order to have a deeper understanding of the plausible roles of this interesting class of genes in HNC carcinogenesis, we first summarize their aberrant expression patterns in HNC per the published reports, followed by analysis of their chromosomal locations in relation to specific exons of the corresponding paired sense genes.

### 2.1. Chromosomal Locations of 52 HNC-Related Antisense Genes in Relation to Respective Sense Gene/Exon(s)

A human antisense gene, transcribed from the antisense orientation of a specific sense gene or exon(s), is believed to have a presumed role in the regulation of the gene or exon of interest. Yet, such a presumed role still needs to be experimentally proven for all antisense genes. To aid future functional validation studies, we listed the detailed chromosomal locations of the 52 HNC-reported antisense genes, the exact lengths and locations in relation to the sense target genes/exon(s), the reported number of antisense gene isoforms (based on UCSC Genome Browser information), and the length ratios of the antisense vs. sense gene in Table 1.

First, the 52 HNC-studied antisense genes have lengths ranging from 989 to 227,501 nucleotides, with the longest being *STARD4-AS1*, and the shortest being *HOXC-AS1*. Second, based on the GenBank and UCSC Genome Browser data, most of these antisense genes have only one isoform, while 19 of them are known to have multiple isoforms (ranging from 2 to 32 isoforms; Table 1). Further, we found that irrespective of the length of the antisense gene, none of these 52 antisense genes cover all exons of the presumed target/corresponding sense gene. In fact, when we compared the ratio of the antisense gene/presumed target gene lengths, 39 such antisense genes were shorter than their sense target genes, among which, 16 of them bear less than 10% of the target gene length. Overall, 13 antisense genes are longer than their corresponding sense genes, with *MSC-AS1* being 74.72 times longer than the corresponding *MSC* gene (*musculin*; a very small human gene with only two exons). *MSC-AS1* resides in the antisense orientation, overlaps with exon 1, the 5′-UTR, and is far ahead of the MSC gene (Table 1). It is likely that the *MSC-AS1* gene, whose length far exceeds that of *MSC*, plays a regulatory role, both locally and beyond. This significant length difference suggests that *MSC-AS1* may regulate *MSC* gene expression, likely by short-range and long-range mechanisms such as chromatin remodeling and transcriptional interference, etc. Interestingly, a closer examination of the nearby genes or enhancers covered by *MSC-AS1* reveals that it overlaps with a major part of the *TRPA1* gene (*transient receptor potential cation channel subfamily A member 1*), with a not-yet-identified function, as well as more than 10 enhancers, including two H3K4me1 hESC enhancers (LOC127459754 and LOC127459755), the BRD4-independent group 4 enhancer (LOC126860417), the MED14-independent group 3 enhancer (LOC126860416), and multiple enhancers with unknown gene interactions, as mapped by a massive ATAC-STARR-seq based on NCBI gene information (www.ncbi.nlm.nih.gov). It is plausible that *MSC-AS1* may have a much wider enhancer-related regulatory function on other genes in addition to *MSC*. This may warrant further investigations.

### 2.2. Antisense Genes on Exon 1, Early Introns, or Promoter Region of Sense Genes

Sixteen of the fifty-two antisense genes (30.8%) have sequence complementarity to the first exon of the corresponding sense genes, which is noteworthy for sense gene regulation. First, exon 1 is usually positioned immediately downstream of the promoter region, where transcription is initiated. Antisense gene transcription (RNA expression) with exon 1 sequence complementarity can significantly influence sense gene expression through promoter interference, chromatin remodeling, and transcriptional collision [1]; or, it can affect the mRNA stability of the sense gene by mediating its degradation [56]. Additionally, many human promoters are inherently bidirectional, meaning that they possess the capacity to initiate transcription in both sense and antisense directions from the same genomic locus. This bidirectional transcription often results in the production of antisense RNAs originating in close proximity to the transcription start site (TSS) of protein-coding genes, frequently leading to overlap with exon 1 or the 5′-UTR of the corresponding sense gene [57,58]. This genomic arrangement allows antisense genes to engage in cis-regulatory mechanisms that modulate the expression of their corresponding sense genes. By overlapping or residing near promoter regions, antisense transcripts can influence promoter activity by altering the accessibility or configuration of transcription factor binding sites. They can also affect the recruitment and progression of RNA polymerase II, thereby impacting transcription initiation and elongation. Furthermore, antisense transcription can induce local histone modifications, such as changes in methylation or acetylation marks, leading to chromatin remodeling that either facilitates or represses gene expression [1,56].

Six of the fifty-two antisense genes (11.5%) have sequence complementarity to intron 1 or intron 2 of the corresponding sense genes. These are as follows: *FAS-AS1*, *FUT8-AS1*, *KIF26B-AS1*, *PRKCA-AS1*, *LHFPL3-AS1*, and *IGF2BP2-AS1*. Introns are mostly known to be involved in splicing; therefore, effects on introns by these antisense genes may likely regulate splicing (thus, altering splice variant expression patterns) and sense gene expression by interference with spliceosomes and RNA polymerase recruitment, respectively [1,59]. Further, recent findings reveal that introns can also increase sense gene expression without serving as binding sites for transcriptional factors; rather, through intron-mediated enhancement mechanisms, they can alter the transcription rate, nuclear export, and transcript stability of the sense gene factors [60], thus regulating the dynamics of sense gene expressions.

Interestingly, 17 of the 52 antisense genes (32.7%) do not overlap with but only reside adjacent to their corresponding sense genes at the genomic level. These include the following: *FGD5-AS1*, *FOXD2-AS1*, *HNF1A-AS1*, *HOXC-AS1*, *HOXA11-AS*, *KTN1-AS1*, *COX10-AS1*, *MNX1-AS1*, *RASAL2-AS1*, *RHPN1-AS1*, *SLC16A1-AS1*, *USP2-AS1*, *VWA8-AS1*, *ZFAS1*, *RGMB-AS1*, *ZNF667-AS1*, and *ELF3-AS1*. It is plausible that the antisense genes residing at the 5′-UTR of the sense genes may affect the promoter or enhancer activity of the adjacent sense genes, while those residing at the 3′-UTR of the sense genes may affect mRNA stability, processing, and localization of the sense genes [61]. Lastly, these antisense genes could potentially exert their regulatory effects through non-sequence complementary mechanisms, such as transcriptional interference, chromatin remodeling, or long-range regulation on the adjacent sense genes and even some other genes nearby as well [60].

### 2.3. Additional HNC-Relevant Antisense Genes Revealed by TCGA-HNC Recurrent Focal Amplified/Deleted Regions

Recently, the Cancer Genome Atlas (TCGA) characterization of HNC patient tumors (N = 279 cases) revealed multiple notable recurrent focal amplified and deleted chromosomal regions in which 27 antisense genes are located [62]. Table 2 lists the location of TCGA-reported HNC recurrent focal amplification or deleted regions bearing 27 residing antisense genes with reported q values in the cohort [62]. Among which, four have been previously studied in HNC (*AFAP1-AS1*, *DLX6-AS1*, *OIP5-AS1*, and *MRPL23-AS1*). Consistent with TCGA data, *DLX6-AS1* expression has been previously reported to be up-regulated in Laryngeal Squamous Cell Carcinoma (LSCC) and nasopharyngeal carcinoma (NPC) tissues [8,63]. However, inconsistency exists for *AFAP1-AS1*, *OIP5-AS1*, and *MRPL23-AS1*, which were reported to reside within the recurrent focal deleted loci by TCGA-HNC cohort; N = 279, yet, they have been previously reported to be frequently upregulated in head and neck tumor tissues, including NPC, oral squamous cell carcinoma (OSCC), tongue squamous cell carcinoma (TSCC), salivary adenoid cystic carcinoma (SACC), and LSCC [3,30,34,64,65,66,67,68]. Future investigations are warranted in larger and more ethnically diverse HNC cohorts as TCGA cohort mainly comprises Caucasian HNC patients.

For the remaining 23 TCGA-associated antisense genes, five of them have been studied in other cancer types. For instance, *DICER-AS1* downregulation was reported to be associated with advanced staging in gastric cancer [69]; *NPPA-AS1* downregulation was associated with tumor aggressiveness in cervical squamous cell carcinoma and endocervical adenocarcinoma (CESC) [70]; and tumor-specific downregulation of *ENTPD3-AS1* was reported to be associated with poorer patient overall survival (OS) and relapse-free survival (RFS) in LUAD [71], followed by the subsequent biological demonstration of ENTPD3-AS1′s suppressive role in LUAD cell growth and migration. Lastly, *BVES-AS1*, which is located at a recurrently deleted genomic region of HNC (TCGA-HNC) [62], has also been reported to be downregulated in colorectal cancer (CRC), with a significant association with TNM staging in CRC patients [72]. Overall, the aberrant downregulation of these antisense genes in the published TCGA-HNC cohort and additional cancer types implicate that certain antisense genes may worth future clinical and biological investigations for biomarker purposes.

## 3. Clinical Significance of 52 Antisense Genes in HNC

### 3.1. Prognostic Findings on Antisense Genes in HNC Patient Cohorts

Since the biological roles of antisense genes (or their transcripts) in humans can be complex and multifaced, cancer researchers often examine their clinical values, mainly by examining their RNA expression (i.e., the “antisense transcripts”) in patient cohorts prior to subsequent functional investigations. As summarized in Table 3, a total of 27 antisense genes/transcripts have been examined for their potential clinical significance in either TCGA-HNC cohort (the largest cohort to date) or independent cohorts. First, 24 such antisense transcripts were found to be overexpressed in HNC patient tumors compared to adjacent normal tissues or normal control tissues, and were reported to be associated with poorer overall survival (OS) or poorer disease-free survival (DFS), implicating their potential involvement in HNC tumorigenesis or progression. Notably, the prognostic significance of *HOXA10-AS*, *LEF1-AS1*, *MSC-AS1*, and *ZEB2-AS1* has been independently reported in multiple cohorts with consistent patient outcome associations and will be discussed at length below. For the antisense genes that predict patient outcomes in TCGA-HNC cohort, we believe that future clinical validations would be important to help classify them as “validated biomarkers” for HNC. One side note, only two antisense transcripts, namely *COX10-AS1* and *SBF2-AS1*, were found to have specific downregulation in HNC and were associated with poorer OS (Table 3).

In the section below, we will focus on four antisense transcripts (*HOXA10-AS*, *LEF1-AS1*, *MSC-AS1*, and *ZEB2-AS1*), which have been cross-validated in multiple independent HNC patient cohorts, and which can be harnessed for future clinical biomarker development with confidence.

*HOXA10-AS* (also known as *HOXA-AS4*) is a relatively novel antisense gene. It is predicted by Alliance of Genome Resources to be involved in miRNA-mediated post-transcriptional gene silencing and is likely part of the RISC complex. As shown in Table 3, independent studies showed that *HOXA10-AS* overexpression was significantly associated with poorer OS in oral and laryngeal SCC patients [18,75,76,80]. Its overexpression was also found to be associated with poorer survival in other malignancies, including low-grade glioma [81], lung adenocarcinoma [82], gastric cancer [83], and leukemia [84]. Using two oral cancer cell lines, SAS and SCC25, it has been demonstrated that the specific knockdown of *HOXA10-AS* mRNA impeded cancer cell growth, migration, and clonogenicity in vitro. Further, overexpression and specific knockdown of *HOXA10-AS* transcript in SAS cells resulted in increased and reduced tumor growth in vivo, respectively, confirming *HOXA10-AS*’s role in promoting oral cancer growth. Bioinformatics analyses showed that *HOXA10-AS* could be linked to *TP63* mRNA processing. Though the potential activity of *HOXA10-AS* on its presumed sense gene has not been studied in HNC, the regulatory activity of *HOXA10-AS* on HOXA10 has been demonstrated in esophageal cancer and glioblastoma models [85,86]. Interestingly, in lung adenocarcinoma models, *HOXA10-AS* was linked to Wnt/beta-catenin signaling, and its expression was regulated by the transcriptional factor ELK1 [82]. In gastric cancer cells, *HOXA10-AS* serves as an oncogene, promoting gastric tumor growth by sponging up *miRNA-6509-5p*, thus enhancing YBX1 signaling for tumorigenic activities [83].

*LEF1-AS1* (also known as *LEF1NAT*) is a known oncogene in glioblastoma, hepatocellular carcinoma, and oral cavity SCC (OSCC) [24,87,88]. Using 88 pairs of OSCC tissues, Zhang et al. showed that *LEF1-AS1* was significantly upregulated in OSCC and such an upregulation was associated with poorer prognosis in patients [77]. This finding was consistent with observations from Fan et al. using TCGA-HNC database [77]. Subsequent functional studies demonstrated that *LEF1-AS1* interacted with LATS1 to suppress Hippo signaling and promote YAP nuclear translocation and function in OSCC [77]. In glioblastoma, *LEF1-AS1* has been demonstrated to act as a competing endogenous RNA (ceRNA) to sponge and downregulate *miR-543*, resulting in the upregulation of EN2 (engrailed homeobox 2) and increased tumor aggressiveness [89]. In hepatocellular carcinoma cells, *LEF1-AS1* can modulate the *miR-10a-5p* level to upregulate MSI1 (Musashi1) expression and activate AKT signaling, contributing to chemoresistance [90]. These findings suggest that *LEF1-AS1* could serve as a prognostic biomarker and therapeutic target in multiple cancer types.

*MSC-AS1* (also known as *MAT1*) is overexpressed in HNC and NPC. Its overexpression was found to be associated with poorer OS in NPC patients and in TCGA-HNC patients [32]. Functional studies showed that *MSC-AS1* promoted NPC cell proliferation, invasion, and epithelial-to-mesenchymal transition (EMT), while inhibiting NPC cell apoptosis. Mechanistically, *MSC-AS1* acts as a molecular sponge for *miR-524-5p*, leading to upregulation of the *NR4A2* oncogene in NPC. In other cancers (gastric and lung cancer), *MSC-AS1* has been shown to have proto-oncogenic roles, including the promotion of cancer cell proliferation, migration, and drug resistance. For example, *MSC-AS1* has been demonstrated to regulate the expression of 6-phosphofructo-2-kinase/fructose-2,6-biphosphatase 3 (FKFB3), thus affecting glycolysis and gastric cancer cell proliferation [91], while in lung cancer cells, it sponges up miR-33b-5ps, thus altering the glycerol-3-phosphate acyltransferase, mitochondrial (GPAM), which has been implied in glycerolipid biosynthesis [92]. In summary, *MSC-AS1* may potentially have diagnostic implications for HNC and some other cancers.

*ZEB2-AS1* (also known as *ZEB2-AS*, *ZEB2AS*, or *ZEB2NAT*) is a naturally occurring antisense transcript located at the 5′-UTR of *zinc finger E-box binding homeobox 2* (*ZEB2*). It is known that activation of the transforming growth factor-beta pathway could drive *ZEB2-AS1* transcription [93]. Diao et al. reported the tumor-specific upregulation of the *ZEB2-AS1* transcript in 71 paired normal–tumor Asian HNC tissues [52]. They reported that patients with tumoral overexpression of the *ZEB2-AS1* transcript had significantly poorer OS and DFS compared to those with low *ZEB2-AS1* expression. Functional data showed that specific knockdown of *ZEB2-AS1* overexpression by the oligonucleotide approach resulted in a reduction in HNC cell growth, cell migration, and clonogenicity, with increased cell death, confirming its tumor-promoting roles in HNC. They further demonstrated that *ZEB2-AS1* could regulate the mRNA stability of its sense gene, *ZEB2*, which is a key transcription factor mediating EMT. In addition to HNC, *ZEB2-AS1* has also been found to be upregulated in hepatocellular carcinoma [94] and bladder [95], lung [96], and gastric cancers [97], etc. With further validations in additional patient cohorts in HNC and other cancers, *ZEB2-AS1* expression may serve as a prognostic biomarker for these cancers.

### 3.2. Clinical Significance of Single Nucleotide Polymorphism (SNP) of Antisense Genes in HNC

In addition to gene upregulation or downregulation, single-nucleotide polymorphisms (SNPs) of several HNC-studied antisense genes have been reported, including those of *CDKN2B-AS1*, *HOTAIR*, and *FAS-AS1*. Here, we summarize the potential clinical importance of their associated SNPs in HNC development.

***CDKN2B-AS1* SNPs:** At least eight *CDKN2B-AS1* SNPs have been reported in various HNC subtypes thus far. In a genome-wide association (GWA) meta-analysis for oral cancer risk, Lesseur et al. identified a susceptibility locus at *CDKN2B-AS1* on 9p21.3. The associated SNP rs8181047 was significantly associated with increased risk of oral cancer in Europe [odds ratio (OR) = 1.18], North America (OR = 1.25), and South America (OR = 1.39), with an overall calculated OR of 1.16 (*p* = 7 × 10^−7^) [98]. However, a subsequent study analyzing five *CDKN2B-AS1* SNPs (rs564398, rs1333048, rs1537373, rs2151280, and rs8181047) in 1060 OSCC cases and 1183 controls in Taiwan found no direct association of rs8181047 with overall OSCC risk, while carriers of a minor allele of rs1333048 (AA changed to CA/CC genotypes) had a higher likelihood of developing advanced-stage OSCC (stage III/IV vs. stage I/II), especially in betel quid chewers and cigarette smokers [99]. There could be potential differences in oral cancer risk associated with rs8181047 among different ethnic groups, which would require further investigations. In EBV-associated NPC, Wang et al. showed that in Southern China, the rs2069418 of *CDKN2B-AS1*, in particular, the CC genotype, was associated with increased NPC risk compared to individuals bearing the heterozygote GC genotype (the cohort comprised 10,472 NPC cases and 6907 control cases). Further, such a CC genotype may promote the expression of *CDKN2B-AS1* in NPC cells by increasing the enhancer activity of CRE3 [100]. Subsequent overexpression and specific gene knockdown experiments demonstrated that *CDKN2B-AS1* likely acts as an oncogene in NPC.

***HOTAIR* SNPs:** A total of six *HOTAIR* SNPs have been investigated in HNC. Su et al. examined the potential association of four *HOTAIR* SNPs, namely rs920778, rs1899663, rs4759314, and rs12427129, in a Taiwanese oral cancer cohort (907 OSCC vs. 1200 control cases). They found that rs1899663 (TT genotype) was significantly associated with an increased risk of OSCC with an adjusted odds ratio (AOR) of 2.227. Among the OSCC cases were 193 non-betel quid users. Subsequent analysis focusing on non-betel quid users’ risk, with rs1899663 (TT genotype) significantly associated with increased OSCC risk with an AOR of 2.380, and AORs of 1.437 and 1.436 for rs920778 (TC and TC + CC genotypes, respectively) [101]. In a Chinese HNC cohort (366 HNC and 732 control cases), *HOTAIR’s* SNP rs4759314 was found to be associated with increased risk of HNC, especially among older individuals and alcohol consumers. rs4759314 was associated with a significantly increased risk of HNC among the Chinese population [GG vs. AA: adjusted odds ratio (OR) = 1.23, 95% confidence interval (CI) = 1.01–1.50; additive model: OR = 1.21, 95% CI = 1.01–1.46]. Stratified analysis showed that this association remained significant among older individuals (adjusted OR = 1.32, 95% CI = 1.01–1.72, *p* = 0.040) and drinkers (adjusted OR = 1.50, 95% CI = 1.02–2.19, *p* = 0.040). No significant associations were observed between rs874945 or rs7958904 and HNC risk [102]. It is plausible that some *HOTAIR* SNPs may contribute to HNC development.

***FAS-AS1* SNP:** In a cohort of 684 NPC and 823 control subjects, Guo et al. reported that individuals carrying the CC genotype of the *FAS-AS1* SNP, rs6586163, were associated with reduced risk of NPC (CC vs. AA genotype, OR = 0.645, *p* = 0.006) and better OS (AC + CC vs. AA genotype, HR = 0.667, *p* = 0.030) compared to those with the AA genotype. Further, it was demonstrated that overexpression of the rs6586163 variant (CC genotype) led to *FAS-AS1* upregulation in NPC cell models, which in turn altered Fas isoform splicing, reduced NPC cell growth, and promoted apoptosis [11].

These reported SNPs of the above antisense genes may have the potential to aid the identification of susceptible individuals for HNC prevention purposes upon future validations in independent cohorts.

## 4. Functional Roles of Antisense Genes/Transcripts in HNC

A subset of the 52 antisense genes has been subjected to some levels of functional studies in HNC models and found to be involved in HNC growth, cell growth, invasiveness, and signaling alteration, as well as drug or radiotherapy responses. Antisense genes with such demonstrated biological functions are listed in Table 4 and discussed below. Most of these functional studies were conducted using overexpression or specific gene knockdown approaches in HNC cell models or animal models.

### 4.1. Proliferation, Cell Cycle, and Cell Death Regulation in HNC

Using overexpression or knockdown approaches (siRNA or shRNA), 39 antisense genes/transcripts have been found to promote HNC cell growth or cell cycle progression or regulate cell death in vitro. In fact, most of these antisense genes have been demonstrated to have potential proto-oncogenic roles in HNC. Except for *FAS-AS1*, *COX10-AS1*, *NR2F2-AS1*, *SLC16A1-AS1*, and *SSTR5-AS1*, their overexpression in HNC cells resulted in the suppression of cell growth, indicative of their potential tumor-suppressive roles in HNC.

Imbalances between cell proliferation and cell death could contribute to tumorigenesis. In addition to promoting cell proliferation, three antisense genes/transcripts have been known to reduce apoptosis in HNC models. These include *BBOX-1-AS1*, *TTN-AS1*, and *MSC-AS1*. Zhao et al. reported that *BBOX-1-AS1* silencing could result in the inhibition of OSCC cell proliferation accompanied by an increase in apoptosis [4]. Fu et al. reported *TTN-AS1* upregulation in OSCC cells and specific knockdown of *TTN-AS1* significantly inhibited cell proliferation with increased apoptosis [47]. Thus, these antisense genes/transcripts could promote HNC tumorigenesis, potentially via the inhibition of apoptosis.

### 4.2. Epithelial–Mesenchymal Transition (EMT), Migration, and Invasion in HNC

EMT is a key process by which epithelial cells gain invasion and metastatic potential via transitioning to a mesenchymal state. Studies have shown that various antisense genes can regulate EMT in HNC cells by modulating the expression and stability of EMT-related genes, including E-cadherin, N-cadherin, vimentin, fibronectin, and matrix-metalloproteases (MMP proteins), etc. It is important to note that EMT-promoting antisense genes are potential targets for anti-metastatic therapy in HNC.

As shown in Table 4, 45 antisense genes/transcripts have been demonstrated to promote EMT or invasiveness of HNC cells. For example, *FUT8-AS1* depletion has been shown to suppress OSCC cell migration and EMT via the modulation of a complex miR-944/FUS/TCF4 (transcription factor 4) feedback loop. Specific knockdown of *FUT8-AS1* resulted in increased expression of E-cadherin and reduced expression of N-cadherin, vimentin, MMP2, and MMP7, which was accompanied by a reduction in cellular migration activities [14].

In OSCC, *HNF1A-AS1* has also been reported to promote cancer cell aggressiveness and EMT, as *HNF1A-AS1* silencing resulted in EMT inhibition in OSCC cells [16]. Similarly, several other antisense genes, including *VIM-AS1*, *ZEB1-AS1*, and *ZEB2-AS1*, have also been shown to promote EMT in OSCC models [49,51,95]. In contrast, *NR2F2-AS1* inhibits TGF-beta-induced EMT, migration, and invasion, as well as inhibiting angiogenesis, indicating its role in EMT suppression in OSCC [33].

In LSCC and NPC, five antisense genes, namely *MSC-AS1*, *KTN1-AS1*, *SBF2-AS1*, *TM4SF19-AS1*, and *ZEB2-AS1*, have been reported to regulate EMT. In particular, *SBF2-AS1* has been shown to suppress EMT and migration of LSCC cells in vitro and LSCC metastasis in vivo. *SBF2-AS1* serves as competing endogenous RNA (ceRNA) to sponge up *miR-302b-3p* (which acts to downregulate TGFBR2 by binding to the 3′-UTR of *TGFBR2*), thus resulting in the upregulation of both *TGFBR2* mRNA and protein in LSCC cells. It was postulated that such an *SBF2-AS1*-mediated TGFBR2 upregulation likely suppresses EMT and migration in LSCC [40]. In contrast to *SBF2-AS1*, the remaining four antisense genes (*MSC-AS1*, *KTN1-AS1*, *TM4SF19-AS1*, and *ZEB2-AS1*) promote EMT, implicating their potential roles in promoting cancer progression. *MSC-AS1* has been shown to be upregulated in NPC tissues and cells. Functional studies showed that *MSC-AS1* could enhance NPC cell invasion and EMT, in addition to its effects on promoting NPC cell proliferation. Mechanistically, *MSC-AS1* acts as a ceRNA, sponging up the tumor-suppressive *miR-524-5p* and resulting in *miR-542-5p* downregulation in NPC cells [32]. Thus, *MSC-AS1* upregulation in NPC could elicit oncogenic effects via downregulating the tumor suppressor *miRNA-542-5p* with its associated target gene effects.

Similarly to *MSC-AS1*, *KTN1-AS1* has also been shown to serve as a ceRNA in HNC, promoting cell proliferation, migration, invasion, and EMT by sponging and depleting *miR-153-3p*, thus resulting in the dysregulation of two EMT genes, *SNAI1* and *ZEB2*, in HNC cells [23].

### 4.3. Signaling Pathway Regulation or Interactions

**Figure 2:**
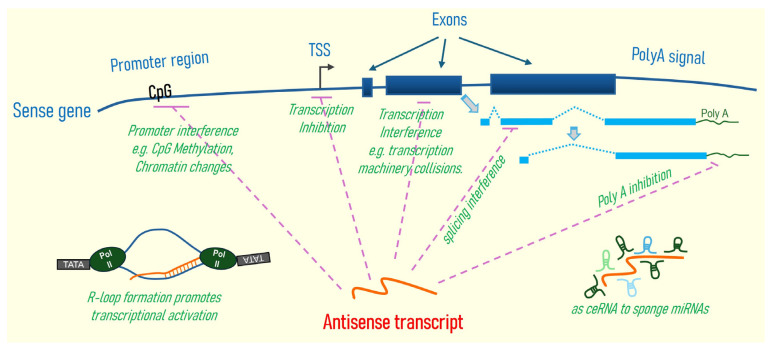
Schematic depicting plausible mechanisms of gene regulation by antisense transcripts. (1) Promoter interference includes interaction of antisense transcript with DNA or DNA methyltransferases, resulting in alteration of CpG methylation status of the sense gene promoter, antisense transcript’s interaction, or recruitment of histone-modifying enzymes, thus altering chromatin structure and epigenetic control of the sense gene [56]. (2) Interference of sense gene transcription by blockade of transcription initiation at the transcription start site (TSS), exons, or even introns of the sense gene via collision of RNA polymerases/transcriptional machineries during sense and antisense gene transcription [1]. In addition, binding of an antisense RNA transcript to sense RNA transcript can also mediate sense RNA transcript degradation. (3) Splicing interference of sense gene transcripts can occur when antisense transcript blocks the access of, for example, splicing factors that are necessary for splicing of nascent sense transcripts. (4) Polyadenylation of sense mRNA transcripts can be affected if the antisense transcript overlaps with such polyA signals of the sense gene [103]. (5) R-loop formation by antisense transcript can promote sense gene transcription. The antisense transcript can form part of a hybrid DNA:RNA structure known as the R loop, which can facilitate the opening of the chromosome to help optimal binding of various transcriptional activators of the sense gene [104]. (6) Antisense transcripts can serve as scaffold ceRNAs (competing endogenous RNAs) competing for microRNAs (miRNAs) in the cell, thus altering the availability of miRNAs and affecting the degradation of a variety of cellular mRNAs. Sense gene transcripts are shown with exons depicted as light blue boxes, and introns with blue dotted line; antisense gene transcripts are depicted as orange curves; purple dotted lines indicate inhibition. TATA boxes, and Polymerase II (Pol II) are shown for R-loop.

Many antisense genes are known to be key regulators of their respective sense genes. Multiple mechanisms could be involved, including the antisense transcripts’ ability to interfere with the sense gene’s transcription by forming RNA-DNA hybrids that would affect sense gene transcription initiation, promoter competition, DNA methylation, and transcriptional interference by collision of RNA polymerases/transcriptional machinery (between the antisense and sense transcription), etc. They could also affect sense mRNA splicing/isoform expressions, protein translation, and even RNA degradation due to their base complementary characteristics with the sense genes/transcripts. Though antisense RNA transcripts usually do not code for proteins, they can contain domains that can interact with DNA, RNA, and even proteins due to their RNA structure/folding flexibility. Thus, they can serve as dynamic scaffolds to bind other DNA, RNA, miRNA, or proteins, and even form functional complexes [1] and alter various other signaling potentials. As illustrated by *SBF2-AS1* above, antisense can serve as a ceRNA, such as *SBF2-AS1* (as discussed above), sponging up the miR-302b-3p responsible for modulating the expression or stability of many mRNAs (thus, proteins), including TGFBR2 and potentially others. Further, if antisense transcripts affect transcriptional factors, such as homeobox transcription factors (e.g., HOXA11), expressions of various target genes of those transcription factors would be affected, which could in turn impact a wide range of signaling events associated with such transcription factors. In summary, antisense transcripts can exhibit their effects on specific sense genes (i.e., in a cis-acting manner) or exert global effects (in a trans-acting manner) on various DNA (e.g., interacting with enhancers or promoters of its own or of other genes), RNA (e.g., affecting RNA stability, splicing, and sponging up miRNAs, etc.), and proteins (e.g., affecting protein stability or function, etc.), thus potentially affecting various signaling pathways in cells. Therefore, it is important to realize that a single antisense transcript could theoretically regulate multiple signaling events, given its wide range of dynamic “molecular switch” effects. A summary of the potential mechanisms of gene regulation by antisense genes/transcripts is graphically depicted in Figure 2.

Though our understanding of the full range effects of all 52 HNC-related antisense genes/transcripts on HNC signaling is minimal, several of them have been shown to regulate or modulate crucial HNC signaling pathways, including the EGFR/MAPK, PI3K/Akt, NF-κB, Notch, TGF-beta, and HIF-1α/VEGF pathways, etc. By regulating key signaling molecules within these pathways, the antisense genes can regulate HNC cell proliferation, invasion, angiogenesis, and even sensitivity to specific inhibitors, etc. Furthermore, we found that 13 out of the 52 antisense genes are known/putative transcription factors (e.g., homeobox proteins and zinc finger proteins) (Table 1); it is likely that they could exert extensive signaling effects in HNC cells by altering multitudes of transcriptional events.

For example, in tongue SCC, *FOXD2-AS1* has been reported to be significantly upregulated in tumor tissues based on TCGA-HNC dataset. Its elevated expression was found to be significantly associated with advanced staging and lymphatic metastasis. Specific knockdown of *FOXD2-AS1* in two cell lines, namely SCC-9 and CAL-27, were found to inhibit p65 expression and phosphorylation (key NF-κB players) and inhibit the MAPK pathway by inhibiting Erk1 phosphorylation [73]. Similarly, Ai et al. showed that *DCST1-AS1* (which promotes HNC migration and invasion) could also regulate NF-κB signaling in HNC cells and in THP-1 differentiated macrophages by downregulating p65 protein expression [7].

Hypoxia-inducible factor 1-alpha (HIF-1α) is an important transcription factor regulating cellular adaptation in hypoxia, a condition that is essentially found in the inner hypoxic core of most human tumors. Recently, Tang et al. showed that *USP2-AS1* was specifically upregulated in HNC cells during hypoxia, and it was a direct transcriptional target of HIF-1α. Further, this hypoxia-inducible *USP2-AS1* could functionally promote HNC cell growth and invasion in vitro, and tumor growth in vivo. It was postulated that *USP2-AS1* might affect E3 ubiquitin ligase DCAF13 expression and, thus, affect the associated signaling in HNC cells [48].

KEAP1 is an important oxidative stress sensor. It is known that KEAP/Nrf2 signaling enables cells to cope with oxidative stress. Zhao et al. showed that *HOXA10-AS* could modulate KEAP/Nrf2 signaling in human HNC xenograft models. They showed that tumors established from AMC-HN-8 cells with *HOXA10-AS* knockdown by shRNA had increased KEAP1 compared to tumors expressing control shRNA, suggesting *HOXA10-AS* as a potential regulator of KEAP1 in HNC [75]. Further, overexpression of *MNX1-AS1* and *LSAMP-AS1* in HNC cells (AMC-HN-8 and Tu177) could result in moderate activation of the PI3K/AKT pathway via Akt phosphorylation [105].

As discussed earlier, ceRNAs, via sponge effects, may play a role in signaling pathway regulation. In HNC cell models, *SBF2-AS1*, *KTN1-AS1*, and *MSC-AS1* antisense transcripts could serve as ceRNAs to sponge up and thus downregulate the expression of *miR-302b-3p*, *miR-153-3p*, and *miR-524-5p*, respectively. Sponge effects of these antisense transcripts require further investigation regarding whether they regulate classic signaling pathways such as the EGFR/MAPK, PI3K/Akt, NF-κB, Notch, TGF-β, and HIF-1α/VEGF pathways, etc., just as other antisense transcripts can also serve as ceRNAs to modulate key signaling events in HNC tumorigenesis.

### 4.4. Modulation of Drug Responses and Radio-Resistance

As some antisense genes can regulate specific signaling events (or multiple signaling events via ceRNA activity), alter mRNA splicing and stability, and regulate expression levels of the sense proteins, it is plausible that they may be able to alter resistance/sensitivity to drugs, especially for targeted therapies in cancer treatment.

EGFR tyrosine kinase inhibitors (TKIs) have minimal clinical activity in HNC patients. Tan et al. reported two clinical exceptional responders to gefitinib (a first-generation EGFR TKI) with the *EGFR* exon 20 (Q787Q) (c.2361G>A; rs10251977) synonymous mutation [9]. Importantly, this EGFR mutation rests on the overlapping genomic sequence of the *EGFR-AS1* gene, and this specific mutation on the *EGFR-AS1* variant can shift the dynamics of *EGFR* mRNA splicing toward the *EGFR* isoform D (rather than isoform A). Such a shift resulted in EGFR pathway activation and EGFR dependency in HNC cells bearing this mutation, thus conferring sensitivity to gefitinib in mutant HNC cells. They postulated that in HNC cells bearing the homozygous A/A genotype for rs10251977 would be more sensitive to EGFR TKI than cells with the G/A or G/G genotypes. It is plausible yet remains to be further tested in larger HNC cohorts, whether such a mutated *EGFR-AS1* status could impact EGFR TKI sensitivity in HNC by altering *EGFR* mRNA spliced isoform balances.

*FOXD2-AS1* (overexpressed in laryngeal SCC) and *LHFPL3-AS1* (overexpressed in OSCC), have been shown to confer cisplatin resistance in HNC models [13,26]. Specifically, Li et al. showed that *FOXD2-AS1* displayed a tumor-specific upregulation (compared to paired adjacent normal; ~2-fold increase; N = 24 cases), and its upregulation was further found to be associated with disease relapse upon cisplatin-based chemotherapy in a small HNC cohort (N = 14 cases, 9 cases with relapse and 5 cases without relapse), which uncovered the direct involvement of *FOXD2-AS1* in mediating cisplatin resistance in HNC. Mechanistic studies with two HNC cell models (Hep-2 and Tu-212) showed that *FOXD2-AS1* overexpression could mediate cisplatin resistance via STAT3-induced stemness in these cells. In fact, *FOXD2-AS1* has also been shown to confer cisplatin resistance in NSCLC cells as well [106]. As for *LHFPL3-AS1*, it has been demonstrated to be upregulated in multiple HNC cell lines (SCC9, CAL27, SCC25, and HSC3 vs. normal NOK cells), as well as in OSCC tissues (N = 51) greater than 2-fold when compared to adjacent normal cell lines. In cisplatin-resistant HNC cells (SCC9-R and CAL27-R), *LHFPL3-AS1* was upregulated for 2.5–3-fold when compared to parental cells, and siRNA-specific knockdown of *LHFPL3-AS1* reversed the cisplatin resistance of these two models in vitro [26]. Wang et al. showed that *LHFPL3-AS1* was upregulated in EBV-positive radioresistant NPC cells, and siRNA targeting of *LHFPL3-AS1* could reverse radioresistance in vitro [107]. Peng et al. showed that *ZFAS1* contributed to intrinsic resistance to low-dose radiation (at 8Gy) in NPC cell models (C666-1 and SUNE-1), as specific siRNA knockdown of *ZFAS1* by siRNA resulted in increased apoptosis upon radiation treatment in vitro, and such resistance could be mediated via *mir-7-5p* [108].

Lastly, it would be important to further explore if other HNC-related antisense genes with demonstrated clinical significance may also affect patient outcomes by driving treatment resistance.

## 5. Conclusions

Four antisense genes, namely *HOXA10-AS*, *LEF1-AS1*, *MSC-AS1*, and *ZEB2-AS1*, have been clinically cross-validated in multiple cohorts, with clear evidence to support clinical biomarker development in HNC. SNPs of several major antisense genes with evidence for HNC risk or outcomes should be further validated in different ethnic groups, for potential global applications in HNC, including desperate EBV-associated NPC, HPV-associated oropharyngeal cancer, and HNCs unrelated to viruses. Lastly, too little is known about their potential mechanisms on HNC pathogenesis, and more signaling investigations should be conducted to help dissect their molecular contributions to HNC carcinogenesis.

## Figures and Tables

**Table 1:** Genomic information of 52 HNC-related antisense genes in relation to corresponding sense genes (based on NCBI GenBank information with GRCh38p.14 reference genome and NCBI RefSeq annotations from https://www.ncbi.nlm.nih.gov/gene/; retrieved on 11 December 2025). NR number is listed below each antisense transcript. Distance to transcription start site (TSS) or transcription termination site (TTS) of the sense gene is listed for each antisense gene graphically. Ratio of antisense/sense lengths = number of nucleotides of antisense gene/number of nucleotides of the corresponding sense gene. n.d.: not determined.

Antisense Gene			Corresponding Sense Gene (Presumed Target Gene)				Ratio of Antisense/Target Gene Size	AS Activity Reported	
Antisense Gene Name	Size (ntds)	No. of Isoforms	Presumed Target Gene/Exon(s)	Size (ntds)	Distance from TSS/TTS of Sense Gene (GRCh38p.14)	Function(s)		Cis-Acting or Trans-Acting	Ref.
***AFAP1-AS1***	24,839	1	*AFAP1*	181,148	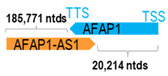	*Actin-filament associated protein 1* (links src or other signaling to actin filament)	0.14	trans-	[3]
*(chr.4p16.1) (NR_026892.1)*			*AFAP1-AS1* overlaps with exons 14–18 of 18 of *AFAP1* (*NM_001134647.2*)						
***BBOX1-AS1***	171,583	3	*BBOX1*	86,995	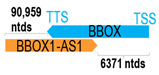	*Gamma-butyrobetaine hydroxylase 1* (catalyzes last step of L-carnitine biosynthesis, essential for mitochondria function)	1.97	trans-	[4]
(*chr.11p14.2-p14.1*) (*NR_125768.1*)			*BBOX-AS1* overlaps with exons 3–9 of 9 of *BBOX1* (*NM_003986.3*)						
***CDKN2B-AS1***	133,352	32	*CDKN2B*	6403	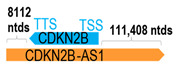	*Cyclin dependent kinase inhibitor 2B* (cell growth and cell cycle)	20.83	trans-	[5]
(*chr.9p21.3*) (*NR_003529.4*)			*CDKN2B-AS1* overlaps with exons 1–2 of 2 of *CDKN2B* (*NM_004936.4*)						
***COX10-AS1*** **(*or COX10-DT*)**	40,167	1	*COX-10*	139,174	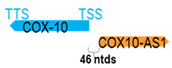	*Cytochrome c oxidase assembly factor heme A:farnesyltransferase COX10* (a heme A:farnesyltransferase required for expression of functional COX)	0.29	trans-	[6]
(*chr.17p12*) (*NR_049718.1*)			*COX10-AS1* is 46 ntds adjacent to *COX10* (*NM_001303.4*)						
***DCST1-AS1***	18,801	2	*DCST1*	17,107	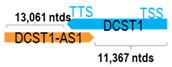	*DC-STAMP domain containing 1* (antigen processing and immune responses)	1.10	n.d.	[7]
(*chr.1q21.3*) (*NR_040773.1*)			*DCST1-AS1* overlaps with exons 11–17 of 17 of *DCST1* (*NM_152494.4*)						
***DLX6-AS1***	45,551	1	*DLX6*	5488	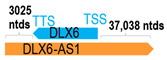	*Distal-less homeobox 6* (involved in forebrain and craniofacial development)	8.30	trans-	[8]
(*chr.7q21.3*) (*NR_015448.1*)			*DLX6-AS1* overlaps with exons 1–3 of 3 of *DLX6* (*NM_005222.4*)						
***EGFR-AS1***	9200	1	*EGFR*	192,612	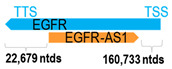	*Epidermal growth factor receptor* (cell proliferation)	0.05	cis-	[9]
(*chr.7p11.2*) (*NR_047551.1*)			*EGFR-AS1* overlaps with exon 20 of 28 of *EGFR-AS1* (*NM_005228.5*)						
***ELF3-AS1***	10,303	1	*ELF3*	6597	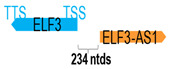	*E74 like ETS transcription factor 3* (involved in inflammatory response and regulation of transcription)	1.56	n.d.	[10]
(*chr.1q32.1*) (*NR_146472.1*)			*ELF3-AS1* is 234 ntds adjacent to *ELF3* (*NM_004433.5*)						
***FAS-AS1***	1554	1	*FAS*	26,262	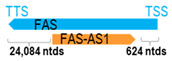	*Fas cell surface death receptor* (regulates apoptosis)	0.06	cis-	[11]
(*chr.10q23.31*) (*NR_028371.1*)			*FAS-AS1* overlaps with intron 1 of *FAS* (*NM_000043.6*)						
***FGD5-AS1***	5663	5	*FGD5*	115,610	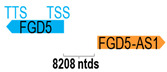	*FYVE*, *RhoGEF, and PH domain containing 5* (actin cytoskeleton organization, filopodium assembly, and regulation of cell shape)	0.05	trans-	[12]
(*chr.3p25.1*) (*NR_046251.1*)			*FGD5-AS1* is 8208 ntds adjacent to *FGD5* (*NM_152536.4*)						
***FOXD2-AS1***	2509	1	*FOXD2*	2648	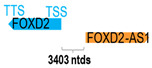	*Forkhead box D2* (belongs to the forkhead family of transcriptional factor, with undetermined function)	0.95	trans-	[13]
(*chr.1p33*) (*NR_026878.1*)			*FOXD2-AS1* is 3403 ntds adjacent to *FOXD2* (*NM_004474.4*)						
***FUT8-AS1***	2026	1	*FUT8*	387,280	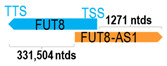	*Fucosyltransferase 8* (catalyzes fucose transfer from GDP-fucose to N-linked type complex glycopeptides)	0.01	trans-	[14]
(*chr.14q23.3*) (*NR_024334.1*)			*FUT8-AS1* overlaps with intron 1 of *FUT8* (*NM_178155.3*)						
***HAS2-AS1***	5980	1	*HAS2*	29,325	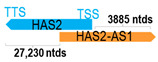	*Hyaluronan synthase 2* (a putative hyaluronan synthase)	0.20	cis-	[15]
(*chr.8q24.13*) (*NR_002835.2*)			*HAS2-AS1* overlaps with exon 1 of 4 of *HAS2* (*NM_005328.3*)						
***HNF1A-AS1***	2455	1	*HNF1A*	23,970	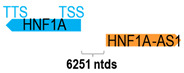	*HNF1 homeobox A* (transcription factor)	0.10	n.d.	[16]
(*chr.12q24.31*) (*NR_024345.1*)			*HNF1A-AS1* is 6251 ntds adjacent to *HNF1A* (*NM_001306179.2*)						
***HOTAIR***	12,643	17	*HOXC11*	4518	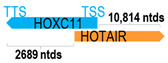	*Homeobox C11* (involved in morphogenesis in multicellular organisms)	2.80	trans-	[17]
(*chr.12q13.13*) (*NR_186241.1*)			*HOTAIR* overlaps with exon 1 of 2 of *HOC11* (*NM_014212.4*)						
***HOXA10-AS***	3017	1	*HOXA10*	3716	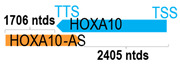	*Homeobox A10* (functions in fertility, embryo viability, and regulation of hematopoietic lineage commitment)	0.81	cis-; trans-	[18]
(*chr.7p15.2*) (*NR_046609.1*)			*HOXA10-AS* overlaps with exon 2 of *HOXA10-AS* (*NM_018951.4*)						
***HOXA11-AS***	3886	1	*HOXA11*	4076	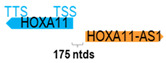	*Homeobox A11* (regulates uterine development and is required for female fertility)	0.95	trans-	[19]
(*chr.7p15.2*) (*NR_002795.2*)			*HOXA11-AS1* is 176 ntds adjacent to *HOXA11* (*NM_005523.6*)						
***HOXC-AS1***	989	1	*HOXC9*	3177	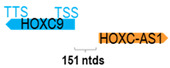	*Homeobox C9* (morphogenesis in multicellular organisms)	0.31	trans-	[20]
(*chr.12q13.13*) (*NR_047504.1*)			*HOXC-AS1* is 151 ntds adjacent to *HOXC9* (*NM_006897.3*)						
***IGF2BP2-AS1***	16,536	1	*IGF2BP2*	181,913	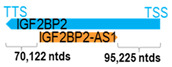	*Insulin-like growth factor 2 mRNA-binding protein 2* (plays an important role in metabolism and gene variants associated with susceptibility to diabetes)	0.09	n.d.	[21]
(*chr.3q27.2*) (*NR_126326.1*)			*IGF2BP2-AS1* overlaps with intron 2 of *IGF2BP2* (*NM_006548.6*)						
***KIF26B-AS1***	28,074	1	*KIF26B*	554,448	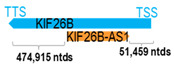	*Kinesin family member 26B* (involved in the transport of organelles along microtubules)	0.05	trans-	[22]
(*chr.1q44*) (*NR_151721.1*)			*KIF26B-AS1* overlaps with intron 2 of *KIF26* (*NM_018012.4*)						
***KTN1-AS1***	3936	1	*KTN1*	104,373	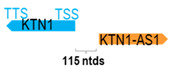	*Kinectin 1* (involved in intracellular organelle motility and the assembly of the elongation factor-1 complex)	0.04	trans-	[23]
(*chr.14q22.3*) (*NR_027123.1*)			*KNT1-AS1* is 115 ntds adjacent to *KNT1* (*NM_001079521.2*)						
***LEF1-AS1***	8906	2	*LEF1*	121,385	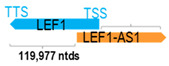	*Lymphoid-enhancer binding factor 1* (enhances activity of T-cell receptor-alpha)	0.07	trans-	[24]
(*chr.4q25*) (*NR_029374.1*)			*LEF1-AS1* overlaps with exon 1 of 12 of *LEF1* (*NM_016269.5*)						
***LEMD1-AS1***	14,189	1	*LEMD1*	40,679	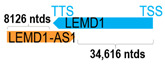	*LEM domain containing 1* (function not yet determined)	0.35	cis-	[25]
(*chr.1q32.1*) (*NR_038425.1*)			*LEMD1-AS1* overlaps with exons 5–6 of 6 of *LEMD1* (*NM_001199050.2*)						
***LHFPL3-AS1***	7587	2	*LHFPL3*	579,959	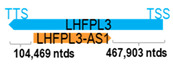	*LHFPL tetraspan subfamily member 3* (belongs to the lipoma HMGIC fusion partner (LHFP) gene family; mutations result in deafness)	0.01	trans-	[26]
(*chr.7q22.2*) (*NR_034141.1*)			*LHFPL3-AS1* overlaps with intron 2 of *LHFPL3* (*NM_199000.3*)						
***LOXL1-AS1***	10,781	5	*LOXL1*	25,675	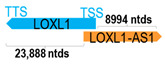	*Lysyl oxidase like 1* (involved in regulation of amine oxidase activity; gene mutation associated with exfoliation syndrome)	0.42	trans-	[27]
(*chr.15q24.1*) (*NR_040068.1*)			*LOXL1-AS1* overlaps with exon 1 of 7 of *LOXL1* (*NM_005576.4*)						
***MACC1-AS1***	11,616	1	*MACC1*	82,730	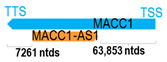	*MET transcriptional regulator MACC1* (regulates HGF receptor pathway, which is involved in proliferation, EMT, invasion and metastasis)	0.14	trans-	[28]
(*chr.7p21.1*) (*NR_046756.1*)			*MACC1-AS1* overlaps with intron 6 of *MACC1* (*NM_182762.4*)						
***MNX1-AS1***	5568	1	*MNX1*	5810	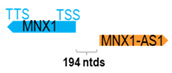	*Motor neuron and pancreas homeobox 1* (mutations result in Currarino syndrome, an autosomal dominant congenital malformation)	0.96	trans-	[29]
(*chr.7q36.3*) (*NR_038835.1*)			*MNX1-AS1* is 194 ntds adjacent to *MNX1*(*NM_005515.4*)						
***MRPL23-AS1***	6712	1	*MRPL23*	67,613	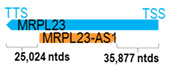	*Mitochondrial ribosomal protein L23* (involved in protein synthesis within the mitochondrion)	0.10	trans-	[30]
(*chr.11p15.5*) (*NR_024471.1*)			*MRPL23-AS1* overlaps with intron 5 of *MRPL23* (*NM_001400176.1*)						
***MSC-AS1***	213,190	2	*MSC*	2853	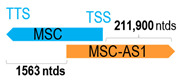	*Musculin* (a transcriptional repressor inhibiting E47’s transactivation capability; also, a downstream target gene of B-cell receptor signaling)	74.72	trans-	[31,32]
(*chr.8q13.3-q21.11*) (*NR_033652.1*)			*MSC-AS1* overlaps with exon 1 of 2 of *MSC* (*NM_005098.4*)						
***NR2F2-AS1***	199,759	3	*NR2F2*	14,213	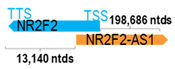	*Nuclear receptor subfamily 2 group F member 2* (a ligand-inducible transcription factor involved in gene regulation)	14.05	trans-	[33]
(*chr.15q26.2*) (*NR_125738.1*)			*NR2F2-AS1* overlaps with exon 1 of 3 of *NR2F2* (*NM_001145155.2*)						
***OIP5-AS1***	30,642	4	*OIP5*	23,319	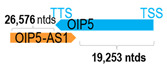	*Opa interacting protein 5* (recruits CENP-A through the mediator Holliday junction recognition protein)	1.31	trans-	[34]
(*chr.15q15.1*) (*NR_152821.1*)			*OIP5-AS1* overlaps with exon 5 of 5 of *OIP5* (*NM_007280.2*)						
***PRKCA-AS1***	18,786	1	*PRKCA*	508,131	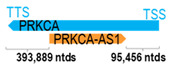	*Protein kinase C alpha* (involved in cell adhesion, cell transformation, cell cycle checkpoint, and cell volume control)	0.04	n.d.	[35]
(*chr.17q24.2*) (*NR_110822.1*)			*PRKCA-AS1* overlaps with intron 2 of *PRKCA* (*NM_002737.3*)						
***RASAL2-AS1***	2486	1	*RASAL2*	384,747	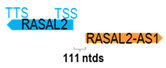	*RAS protein activator like 2* (a GTPase-activating protein activating Ras superfamily of small GTPases)	0.01	trans-	[36]
(*chr.1q25.2*) (*NR_027982.1*)			*RASAL2-AS1* is 111 ntds adjacent to *RASAL2* (*NM_170692.4*)						
***RBM5-AS1***	1386	1	*RBM5*	30,103	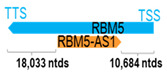	*RNA binding motif protein 5* (involved in the induction of cell cycle arrest and apoptosis through pre-mRNA splicing of multiple target genes)	0.05	trans-	[37]
(*chr.3p21.31*) (*NR_045388.1*)			*RBM5-AS1* overlaps with exons 5–6 of 25 of *RBM5* (*NM_005778.4*)						
***RGMB-AS1***	3467	1	*RGMB*	22,832	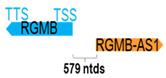	*Repulsive guidance molecule BMP co-receptor b* (contributes to the patterning of the developing nervous system)	0.15	trans-	[38]
(*chr.5q15*) (*NR_033932.1*)			*RGMB-AS1* is 579 ntds adjacent to *RGMB* (*NM_001366508.1*)						
***RHPN1-AS1***	2013	1	*RHPN1*	15,346	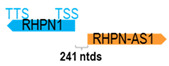	*Rhophilin Rho GTPase binding protein 1* (predicted to regulate stress fiber/focal adhesion assembly, glomerular filtration, and renal albumin absorption)	0.13	trans-	[39]
(*chr.8q24.3*) (*NR_026785.1*)			*RHPN1-AS1* is 241 ntds adjacent to *RHPN1-AS1* (*NM_052924.3*)						
***SBF2-AS1***	53,027	1	*SBF2*	515,552	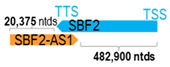	*SET binding factor 2* (encodes a pseudophosphatase and member of the myotubularin-related protein family)	0.10	trans-	[40]
(*chr.11p15.4*) (*NR_036485.1*)			*SBF2-AS1* overlaps with exons 32–41 of 41 of *SBF2-AS1* (*NM_001386339.1*)						
***SLC16A1-AS1***	7658	2	*SLC16A1*	44,350	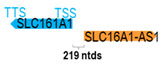	*Solute carrier family 16 member 1* (a proton-linked monocarboxylate transporter catalyzes membrane transport of monocarboxylates, e.g., lactate and pyruvate)	0.17	n.d.	[41]
(*chr.1p13.2*) (*NR_103743.1*)			*SLC16A1-AS1* is 219 ntds adjacent to *SLC16A1* (*NM_003051.4*)						
***SSTR5-AS1***	14,651	1	*SSTR5*	8708	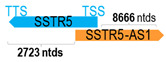	*Somatostatin receptor 5* (G-protein coupled receptor inhibiting adenylate cyclase, and other signaling; mutations result in somatostatin analog resistance)	1.68	cis-	[41]
(*chr.16p13.3*) (*NR_027242.1*)			*SSTR5-AS1* overlaps with exon 1 of 2 of *SSTR5* (*NM_001172560.3*)						
***ST7-AS1***	1889	1	*ST7*	276,676	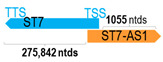	*Suppression of tumorigenicity 7* (function not yet determined)	0.01	trans-	[42]
(*chr.7q31.2*) (*NR_002330.1*)			*ST7-AS1* overlaps with exon 1 of 16 of *ST7* (*NM_001369598.1*)						
***STARD4-AS1***	227,501	1	*STARD4*	16,503	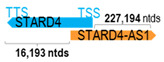	*StAR-related lipid transfer domain containing 4* (cholesterol homeostasis)	13.79	n.d.	[43]
(*chr.5q22.1*) (*NR_040093.1*)			*STARD4-AS1* overlaps with exon 1 of 6 of *STARD4* (*NM_139164.3*)						
***TFAP2A-AS1***	3852	1	*TFAP2A*	18,398	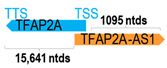	*Transcription factor AP-2 alpha* (functions as either a homodimer or heterodimer with similar family members to inhibit transcription)	0.21	cis-; trans-	[44]
(*chr.6p24.3*) (*NR_033910.1*)			*TFAP2A-AS1* overlaps with exon 1 of 7 of *TFAP2A* (*NM_001372066.1*)						
***TM4SF19-AS1***	7239	2	*TM4SF19*	14,842	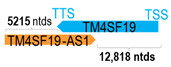	*Transmembrane 4 L six family member 19* (cell proliferation, motility, and adhesion via their interactions with integrins)	0.49	trans-	[45]
(*chr.3q29*) (*NR_046724.1*)			*TM4SF19-AS1* overlaps with exon 4–5 of 5 of *TM4SF19* (*NM_138461.4*)						
***TMPO-AS1***	3254	1	*TMPO*	20,062	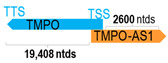	*Thymopoietin* (regulates gene expression, chromatin organization, replication, and cell cycle control)	0.16	n.d.	[46]
(*chr.12q23.1*) (*NR_027157.1*)			*TMPO-AS1* overlaps with exon 1 of 4 of *TMPO* (*NM_003276.2*)						
***TTN-AS1***	97,027	2	*TTN*	281,435	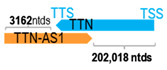	*Titin* (assembly of contractile machinery in muscle cells)	0.34	trans-	[47]
(*chr.2q31.2*) (*NR_038271.1*)			*TTN-AS1* overlaps with exons 249–363 of 363 of *TTN* (*NM_001267550.2*)						
***USP2-AS1***	117,456	1	*USP2*	26,476	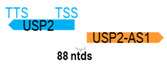	*Ubiquitin-specific peptidase 2* (ubiquitin-specific protease required for TNF-alpha-induced NF-kB signaling)	4.44	n.d.	[48]
(*chr.11q23.3*) (*NR_034160.1*)			*UPS2-AS1* is 88 ntds adjacent to *USP2* (*NM_004205.5*)						
***VIM-AS1***	15,747	2	*VIM*	9353	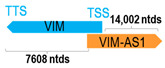	*Vimentin* (EMT, cell attachment, migration, neuritogenesis, and cholesterol transport)	1.68	n.d.	[49]
(*chr.10p13*) (*NR_108061.1*)			*VIM-AS1* overlaps with exons 1–2 of 10 of *VIM* (*NM_003380.5*)						
***VWA8-AS1***	20,397	1	*VWA8*	394,275	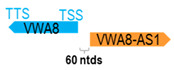	*Von Willebrand factor A domain containing 8* (predicted to enable ATP hydrolysis activity)	0.05	n.d.	[50]
(*chr.13q14.11*) (*NR_039974.1*)			*VWA8-AS1* is 60 ntds adjacent to *VWA8* (*NM_015058.2*)						
***ZEB1-AS1***	11,379	6	*ZEB1*	211,388	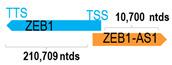	*Zinc finger E-box binding homeobox 1* (transcriptional factor; it may repress interleukin 2)	0.05	trans-	[51]
(*chr.10p11.22*) (*NR_148978.1*)			*ZEB1-AS1* overlaps with exon 1 of 13 of *ZEB1* (*NM_001323638.2*)						
***ZEB2-AS1***	1286	1	*ZEB2*	136,039	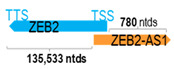	*Zinc finger E-box binding homeobox 2* (functions as a DNA-binding transcriptional repressor interacting with activated SMADs)	0.01	cis-; trans-	[52]
(*chr.2q22.3*) (*NR_040248.2*)			*ZEB2-AS1* overlaps with exon 1 of 10 of *ZEB2* (*NM_014795.4*)						
***ZFAS1***	11,083	5	*ZNFX1*	32,158	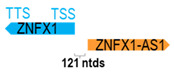	*Zinc finger NFX1-type containing 1* (involved in defense response to bacteria and viruses)	0.34	n.d.	[53]
(*chr.20q13.13*) (*NR_003604.3*)			*ZNAS1* is 121 ntds adjacent to *ZNFX1* (*NM_021035.3*)						
***ZNF667-AS1***	17,564	2	*ZNF667*	38,017	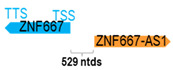	*Zinc finger protein 667* (predicted to regulate transcription by RNA polymerase II)	0.46	cis; trans-	[54,55]
(*chr.19q13.43*) (*NR_036521.1*)			*ZNF667-AS1* is 529 ntds adjacent to *ZNF667* (*NM_022103.4*)						

**Table 2:** TCGA-reported recurrent focal amplified and deleted chromosomal regions with antisense genes located within the detected peak boundaries (peaks defined by GISTIC 2.0 version; data extracted from Cancer Genome Atlas 2015 [62]; N = 279 cases).

Recurrent Focal Copy Change (TCGA 2015; N = 279)	Cytoband	q Value	Wide Peak Boundaries	Antisense Genes Reported Within Peak Boundaries
**Recurrent Focal Amp.**	7q22.1	1.91 × 10^−5^	chr7:96292899-100656111	*DLX6-AS1*
**Recurrent Focal Del.**	1p36.13	2.09 × 10^−5^	chr1:7829287-27191410	*NPPA-AS1*
				*ENO1-AS1*
	2q36.2	8.77 × 10^−14^	chr2:206480055-243199373	*BOK-AS1*
	3p21.1	0.004465	chr3:24020567-61547330	*ENTPD3-AS1*
				*BSN-AS2*
	4p16.2	0.064529	chr4:1-36070348	*AFAP1-AS1*
				*HTT-AS1*
	6q12	0.1766	chr6:64293011-132619682	*AKIRIN2-AS1*
				*BVES-AS1*
				*TRAF3IP2-AS1*
	11p15.4	0.066999	chr11:1-30035954	*IGF2-AS1*
				*NAV2-AS4*
				*BDNF-AS1*
				*MRVI1-AS1*
				*MRPL23-AS1*
	13q21.33	0.000153	chr13:32971238-73359691	*TSC22D1-AS1*
				*TPT1-AS1*
	14q32.32	0.008632	chr14:81998189-107349540	*FAM181A-AS1*
				*ITPK1-AS1*
				*DICER1-AS1*
	15q15.1	0.17007	chr15:1-88420882	*FBXO22-AS1*
				*OIP5-AS1*
	19p13.3	1.90 × 10^−9^	chr19:1-2241326	*CIRBP-AS1*
				*CSNK1G2-AS1*
	19q13.43	0.047985	chr19:57166834-59128983	*A1BG-AS1*
				*PEG3-AS1*

**Table 3:** Specific upregulation or downregulation of antisense RNA transcripts with significance association with head and neck cancer patient outcomes from various cohorts. Published Hazard Ratios (HRs) for overall survival were extracted from the published references. N.A.: not available.

Expression in HNC Tumors	Antisense Gene	Clinical Significance	*p* Value	Reported Hazard Ratio (HR) for OS	Reference
**Upregulated**	***CDKN2B-AS1***	Poorer OS in laryngeal SCC patients (N = 60)	<0.05	N.A.	[5]
	***FOXD2-AS1***	Poorer OS in HNSCC patients (N = 499; TCGA)	<0.05	1.29 (1.13–2.19)	[13]
		Poorer OS in stage IV HNSCC patients (N = 259; TCGA)	<0.05	1.5 (1.06–2.13)	[13]
		Poorer OS in tongue SCC patients (N = 119; TCGA)	<0.05	N.A.	[73]
		Poorer OS in OSCC patients (N = 330; TCGA)	<0.01	1.56 (1.12–2.16)	[74]
		Poorer DSS in OSCC patients (N = 330; TCGA)	<0.01	1.74 (1.15–2.64)	[74]
	***HNF1A-AS1***	Poorer OS in OSCC patients (N = 62)	<0.05	N.A.	[16]
	***HOTAIR***	Poorer OS in OSCC patients (N = 76)	<0.01	N.A.	[17]
		Poorer DFS in OSCC patients (N = 76)	<0.05	N.A.	[17]
	***HOXA10-AS***	Poorer OS in OSCC patients (N = 83)	<0.01	N.A.	[18]
		Poorer OS in laryngeal SCC patients (N = 111; TCGA)	<0.05	1.84 (1.02–3.32)	[75]
		Poorer OS in OSCC patients (N = N.A.; TCGA)	<0.05	1.51	[76]
	***HOXC-AS1***	Poorer OS in nasopharyngeal carcinoma patients (N = 90)	<0.05	N.A.	[20]
	***HOXA11-AS***	Poorer OS in OSCC patients (N = 50)	<0.01	N.A.	[19]
	***LEF1-AS1***	Poorer OS in HNSCC patients (N = 502; TCGA)	<0.05	1.4 (1.06–1.85)	[24]
		Poorer OS in OSCC patients (N = 88)	<0.05	N.A.	[77]
	***MRPL23-AS1***	Poorer OS in ASCC patients (N = 143)	<0.05	N.A.	[30]
	***MNX1-AS1***	Poorer OS in laryngeal SCC patients (N = 40)	<0.05	N.A.	[29]
	***MSC-AS1***	Poorer OS in HNSCC patients (N = 517; TCGA)	<0.01	1.5	[32]
		Poorer OS in NPC patients (N = 34)	<0.01	N.A.	[32]
		Poorer OS in laryngeal SCC patients (N = 517; TCGA)	<0.01	1.5	[31]
	***OIP5-AS1***	Poorer OS in nasopharyngeal carcinoma patients (N = 105)	<0.01	N.A.	[34]
	***RASAL2-AS1***	Poorer OS in HNSCC patients (N = 511; TCGA)	<0.05	1.4	[36]
	***SLC16A1-AS1***	Poorer OS in OSCC patients (N = 359; TCGA)	<0.05	1.4	[41]
	***STARD4-AS1***	**Better OS in OSCC patients (N = 305; TCGA)**	<0.01	N.A.	[43]
		**Better OS in HNSCC patients (N = 500; TCGA)**	<0.01	0.66 (0.50–0.86)	[78]
	***ST7-AS1***	Poorer OS in laryngeal SCC patients (N = 106)	<0.01	N.A.	[42]
	***USP2-AS1***	Poorer OS in HNSCC patients (N = 511; TCGA)	<0.05	N.A.	[48]
	***ZEB1-AS1***	Poorer OS in OSCC patients (N = 88)	<0.05	N.A.	[51]
	***ZEB2-AS1***	Poorer OS in HNSCC patients (N = 71)	<0.05	N.A.	[52]
		Poorer DFS in HNSCC patients (N = 71)	<0.05	N.A.	[52]
		Poorer OS in laryngeal SCC patients (N = 45)	<0.05	N.A.	[79]
	***ZFAS1***	Poorer OS in HNSCC patients (N = 260; TCGA)	<0.05	N.A.	[53]
	***RGMB-AS1***	Poorer OS in laryngeal SCC patients (N = 49)	<0.05	N.A.	[38]
		Poorer DFS in laryngeal SCC patients (N = 49)	<0.05	N.A.	[38]
	***RBM5-AS1***	Poorer OS in OSCC patients (N = 80)	<0.01	N.A.	[37]
	***LHFPL3-AS1***	Poorer OS in OSCC patients (N = 51)	<0.05	N.A.	[26]
		Poorer DFS in OSCC patients (N = 51)	<0.05	N.A.	[26]
	***IGF2BP2-AS1***	Poorer OS in OSCC patients (N = 340; TCGA)	<0.01	N.A.	[21]
**Downregulated**	***COX10-AS1***	Poorer OS in OSCC patients (N = 80)	<0.0001	N.A.	[6]
	***SBF2-AS1***	Poorer OS in laryngeal SCC patients (N = 86)	<0.001	N.A.	[40]

**Table 4:** Functional roles of antisense genes/transcripts in HNC as demonstrated by experimental evidence.

Biological Function	Antisense Genes/Transcripts
HNC Proliferation, Cell Cycle, and Cell Death Regulation	*AFAP1-AS1*, *BBOX1-AS1*, *CDKN2B-AS1*, *DCST1-AS1*, *FAS-AS1*, *HAS2-AS1*, *HNF1A-AS1*, *HOTAIR*, *HOXA10-AS*, *KIF26B-AS1*, *KTN1-AS1*, *LEF1-AS1*, *COX10-AS1*, *LOXL1-AS1*, *MNX1-AS1*, *MSC-AS1*, *NR2F2-AS1*, *OIP5-AS1*, *PRKCA-AS1*, *RASAL2-AS1*, *RHPN1-AS1*, *SLC16A1-AS1*, *SSTR5-AS1*, *STARD4-AS1*, *ST7-AS1*, *TFAP2A-AS1*, *TM4SF19-AS1*, *TMPO-AS1*, *TTN-AS1*, *USP2-AS1*, *ZEB1-AS1*, *ZEB2-AS1*, *ZFAS1*, *RGMB-AS1*, *RBM5-AS1*, *ZNF667-AS1*, *LHFPL3-AS1*, *IGF2BP2-AS1*, *ELF3-AS1*
Epithelial–Mesenchymal Transition (EMT), Migration, and Invasion	*AFAP1-AS1*, *BBOX1-AS1*, *CDKN2B-AS1*, *CDKN2B-AS1*, *DCST1-AS1*, *FGD5-AS1*, *FOXD2-AS1*, *FUT8-AS1*, *HNF1A-AS1*, *HOTAIR*, *HOXC-AS1*, *HOXA11-AS*, *HOXA10-AS*, *KIF26B-AS1*, *KTN1-AS1*, *LEF1-AS1*, *LEMD1-AS1*, *COX10-AS1*, *LOXL1-AS1*, *MNX1-AS1*, *MSC-AS1*, *NR2F2-AS1*, *OIP5-AS1*, *PRKCA-AS1*, *RASAL2-AS1*, *RHPN1-AS1*, *SBF2-AS1*, *SSTR5-AS1*, *STARD4-AS1*, *ST7-AS1*, *TFAP2A-AS1*, *TM4SF19-AS1*, *TMPO-AS1*, *USP2-AS1*, *VIM-AS1*, *VWA8-AS1*, *ZEB1-AS1*, *ZEB2-AS1*, *ZFAS1*, *RGMB-AS1*, *RBM5-AS1*, *ZNF667-AS1*, *LHFPL3-AS1*, *IGF2BP2-AS1*, *ELF3-AS1*
Signaling Interaction	*HAS2-AS1*, *COX10-AS1*, *MACC1-AS1*, *FOXD2-AS1*, *DCST1-AS1*, *USP2-AS1*, *MNX1-AS1*, *HOXA10-AS*, *HOTAIR*, *LEF1-AS1*
Modulation of Drug Responses and Radio resistance	*EGFR-AS1*, *FOXD2-AS1*, *HOTAIR*, *HOXA10-AS*, *MRPL23-AS1*, *ZEB1-AS1*, *ZFAS1*, *LHFPL3-AS1*

## Data Availability

Not applicable. All data were extracted from the original articles cited.

## Supplementary Materials

The following supporting information can be downloaded at: https://www.mdpi.com/article/10.3390/cells15010009/s1, Supplementary Table S1. All annotated antisense genes approved by the HUGO Gene Nomenclature Committee (https://www.genenames.org/; accessed on 13 May 2025).
